# Computer-aided design and 3D printing for a stable construction of segmental bone defect model in Beagles: a short term observation

**DOI:** 10.1186/s41205-024-00217-y

**Published:** 2024-06-25

**Authors:** Kai Cheng, Haotian Zhu, Yuanhao Peng, Xinghua Wen, Huanwen Ding

**Affiliations:** 1 Department of Orthopedics, Guangzhou First People’s Hospital, Guangzhou, 510180 China; 2 School of Medicine, South China University of Technology, Guangzhou, 510006 China; 3 School of Biomedical Sciences and Engineering, South China University of Technology, Guangzhou, 511442 China

**Keywords:** Beagle dog, Animal model, Segmental bone defect, Computer-aided design, 3D printing

## Abstract

**Objective:**

Segmental bone defect animal studies require stable fixation which is a continuous experimental challenge. Large animal models are comparable to the human bone, but with obvious drawbacks of housing and costs. Our study aims to utilize CAD and 3D printing in the construction of a stable and reproducible segmental bone defect animal mode.

**Methods:**

CAD-aided 3D printed surgical instruments were incorporated into the construction of the animal model through preoperative surgical emulation. 20 3D printed femurs were divided into either experimental group using 3D surgical instruments or control group. In Vitro surgical time and accuracy of fixation were analysed and compared between the two groups. A mature surgical plan using the surgical instruments was then utilized in the construction of 3 segmental bone defect Beagle models in vivo. The Beagles were postoperatively assessed through limb function and imaging at 1, 2 and 3 months postoperatively.

**Results:**

In vitro experiments showed a significant reduction in surgical time from 40.6 ± 14.1 (23–68 min) to 26 ± 4.6 (19–36 min) (*n* = 10, *p* < 0.05) and the accuracy of intramedullary fixation placement increased from 71.6 ± 23.6 (33.3–100) % to 98.3 ± 5.37 (83–100) %, (*n* = 30, *p* < 0.05) with the use of CAD and 3D printed instruments. All Beagles were load-bearing within 1 week, and postoperative radiographs showed no evidence of implant failure.

**Conclusion:**

Incorporation of CAD and 3D printing significantly increases stability, while reducing the surgical time in the construction of the animal model, significantly affecting the success of the segmental bone defect model in Beagles.

## Introduction

 The incidence of bone defects resulting from numerous pathologies is gradually increasing with a relatively low clinical cure rate and high rate of disability. Studies on bone tissue engineering however have provided numerous potential treatment options and brought the hope of improving treatment odds. Presently, most studies involving bone tissue engineering showing promising biomaterials still need extensive testing to complete preclinical experiments before reaching the clinical setting leading to the need in animal studies. Bone defect animal models are developing rapidly, and thus the need of a reliable yet reproducible animal model is in constant demand. These models require the selected animals to be physiologically as similar to humans as possible, as well as having a feasible perioperative management protocol and surgical procedure [1]. Commonly used experimental animals for bone defect models include mice, rats, rabbits, dogs, sheep and pigs [2, 3], operated at the parietal bone, mandible, ulna, radius, femur and tibia to emulate a clinical bone defect model [4]. To our knowledge, there is limited segmental weight-bearing bone defect studies in large animals as compared to their smaller counterparts due to numerous challenges. The main issue lies in the relatively complex surgical procedure leading to poor repeatability and the difficulty in ensuring a consistent length of osteotomy which interferes with the subsequent research results. Moreover, there is only a relatively small selection of animals that could tolerate a large segmental weight-bearing site bone defect, and there is a lack of consensus in the most appropriate selection of fixation to support the weight-bearing site after bone defect modelling.

Studies have reported that the anatomical structure and histological features of the Beagle femur highly resemble the structures in humans. Beagles are highly favoured in the experimental setting due to their moderate size, higher levels of compliance, lower levels of pain perception, convenient breeding and immunity compared to other breeds of dogs. Therefore, Beagles are highly suitable to be used as a segmental bone defect animal model [5, 6]. Construction of a segmental bone defect model should conform with the definition of critical bone defects, which is defined as a defect in longitudinal length at least 1.5 times the diameter of the cortical bone [7]. The choice of fixation modality is mainly divided as either use internal or external fixation [8, 9]. External fixation has a relatively higher incidence of infection due to exposed incisions and uncooperative animals. The most common intramedullary nailing system on the other hand has some obvious advantages: (i) the mechanical axis of the operated limb can be adjusted to prevent stress concentrated on one particular region of the operated bone and reduce the risk of refracture; (ii) a closed incision and implantation can preserve periosteal blood supply, reduce the risk of postoperative infection, and retain growth factors during hematoma formation of fracture repair [10–12].

In our study, we incorporated CAD and 3D printing to ensure uniform osteotomy and precision surgery. With the construction of appropriate surgical instruments and preoperative surgical simulation, we aim to provide a fresh insight on a stable segmental weight-bearing bone defect animal model for both bone tissue engineering and eventual clinical use.

## Materials and methods

### Preparation of animals and surgical tools

Three 1-year-old male Beagles with an average body weight of 10.8 ± 1.48 (9.2–12.1) kg were used in this study. All experimental animals were operated and raised by the same group of researchers, and was approved by the Ethics Committee of our institute (BLINDED) before the experiment. All participants of the study have been trained and qualified for animal experimentation.

**Fig. 1:**
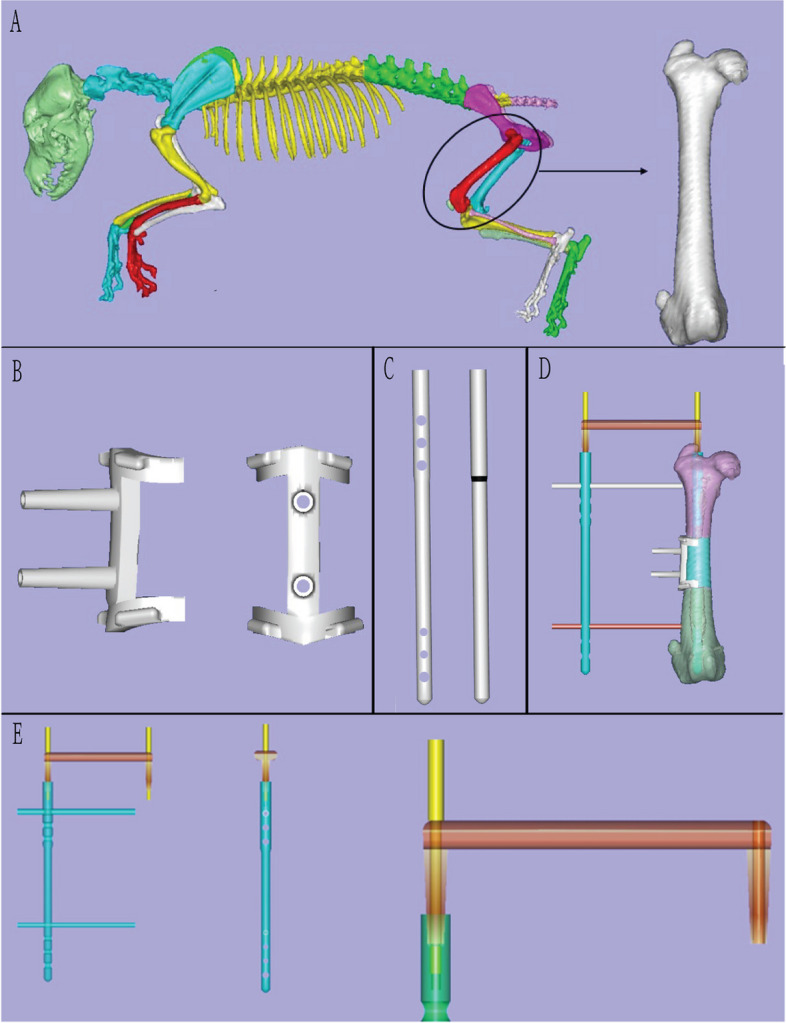
CAD construction model: **A** Three-dimensional Beagle femur model. **B** Osteotomy guide plate; **C** Intramedullary nail; **D**/**E** Intramedullary nail guide system

**Fig. 2:**
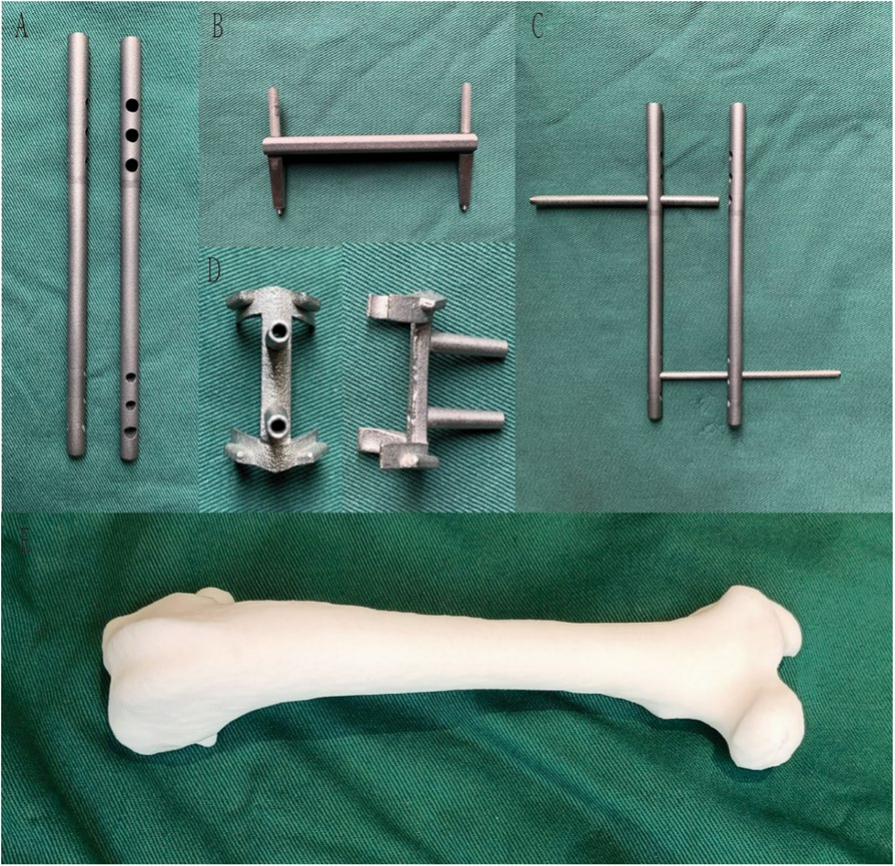
3D printed surgical instruments and Beagle femur model: **A** Intramedullary nails; **B**/**C** Surgically used guide plates acting as a guidance system; **D** Osteotomy guide plates; **E** Resin-based Beagle femur model

All 3 Beagles were anesthetized for CT scanning during the beginning of the study. The resulting DICOM data was obtained and input into Mimics 22.0 (Materialize Software, Leuven, Belgium, USA) and analysed under edit and region grow to obtain the femoral parameters (length of femur, diameter of middle diaphysis and medullary cavity). Femoral data then underwent reverse engineering using Imageware 14.0 (UGS Corporation, Plano, Texas, USA) to design the osteotomy guide plates and intramedullary nails used for each experiment. To facilitate intramedullary nailing insertion, a guide system was further designed and the output was saved in STL format (Fig. 1). Through Finite Element Analysis (FEA), the intramedullary nail was further analysed to assess the stress distribution of the bony structure following insertion of the intramedullary and locking nails. The additional stress applied due to the defect model was equivalent to 1/4 of the average Beagle body weight. Understanding the stress distribution of the femoral bone ensured adequate mechanical strength after surgery. The surgical instruments used in the study were printed by metal melting laser sintering (M290 EOS Germany) and the 20 Beagle femur models were printed using stereolithography (STL) (J750 Strarasys American) with an original ratio of 1:1 (Fig. 2).

### In vitro simulated surgical procedures and evaluation

**Fig. 3:**
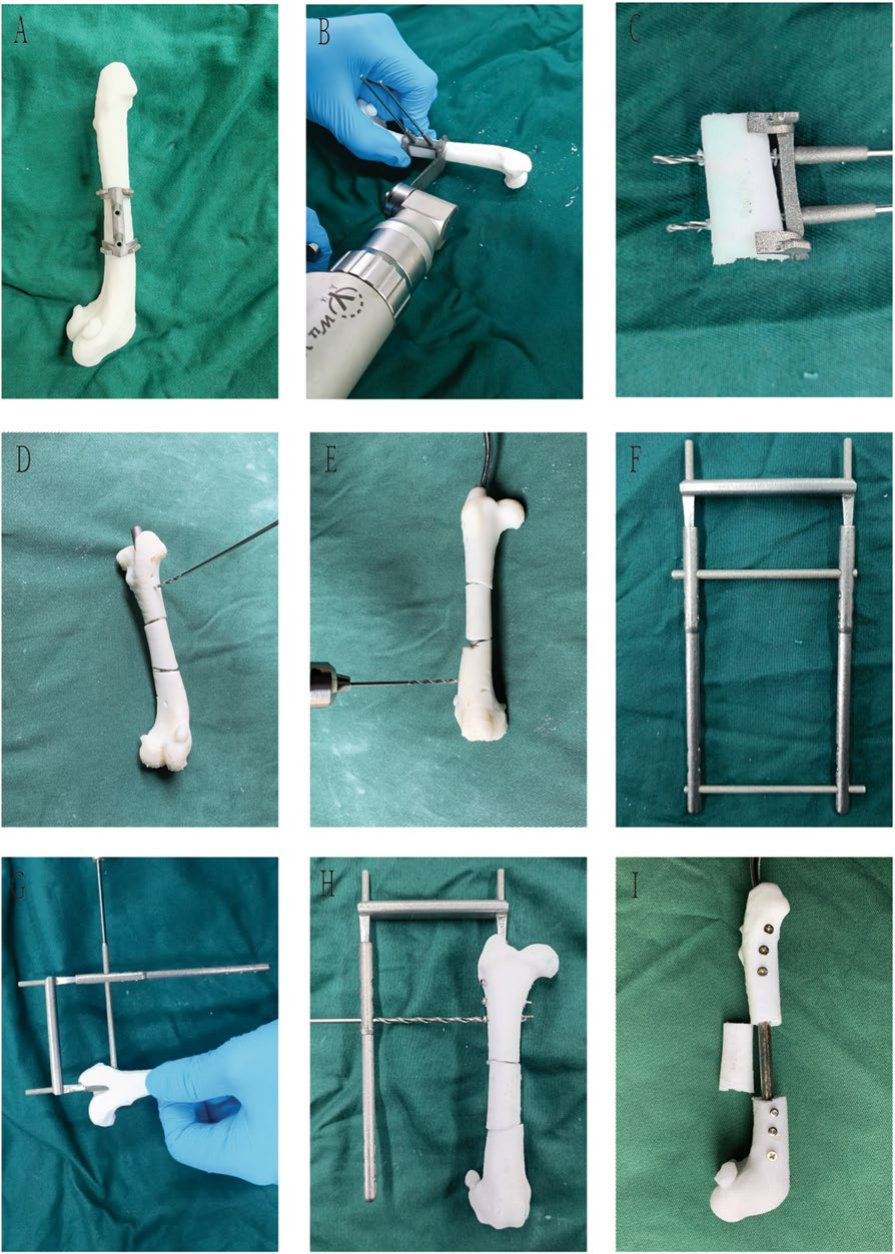
In vitro surgical procedures: **A** Placement of osteotomy guide plate; **B** Osteotomy process; **C** Bone segment amputation; **D**/**E** Drilling by visual method; **F** Personalized oriented system assembled; **G**/**H** Guided drilling; **I** Nail placement is complete

Twenty printed femur models were divided into either operation using the 3D printed surgical instruments experimental group or without additional instruments as the control groups (Fig. 3). The efficacy of CAD was evaluated by comparing the operation time and accuracy of nail placement between the two groups. The femur models underwent the following procedure: (Control group) Following osteotomy of the mid-femur, a standard sized intramedullary nail was inserted into the printed model and marked on the different sites of locking nail positions using a coloured marker. The marked locations were drilled to make an orifice and the locking nails were inserted into each orifice and through the intramedullary nail. (Experimental group) Following osteotomy in the mid-section of the femur and insertion of the intramedullary nail into the model, a surgical guide plate was placed upon the proximal and distal ends and drilled into each protrusion of the guide plate.

**Fig. 4:**
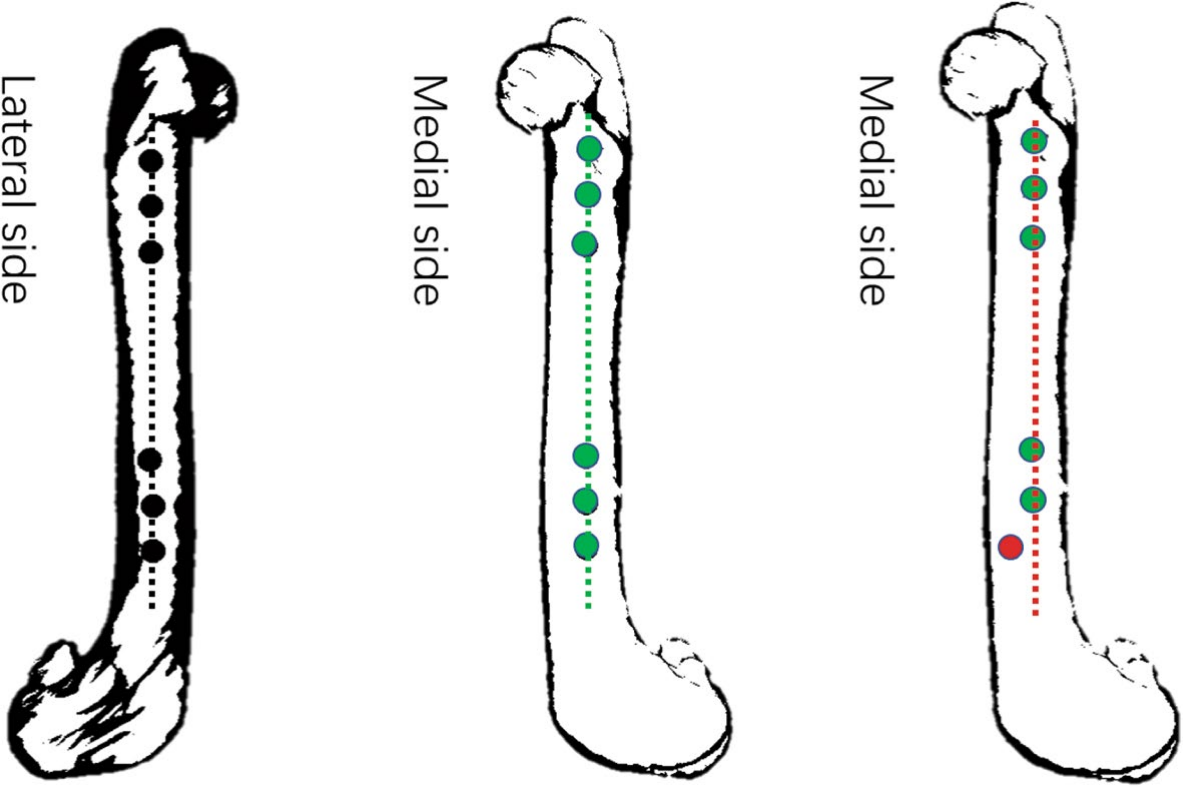
Demonstration of screw insertion accuracy: Black dots represent nail heads, green dots represent accurately positioned nail tails, and red dots represent inaccurately positioned nail tails

The duration of operation was calculated from the start of osteotomy up to the complete implantation of all the locking nails. Accuracy is determined by whether the direction of the drilled orifice is directly perpendicular to the axis of the intramedullary nail (upon successful locking nail insertion, all the nail heads are aligned; if the shank deviates significantly from the orifice, the insertion is regarded as a failure). A representation of the procedure is shown in Fig. 4.

### In vivo surgical procedures and evaluation

Canines in our study were firstly evaluated to rule out dermatological problems and assessed for physical mobility, vital signs and general health. Three 1-year old beagles were selected for our in vitro experiment. A control group was not set due to considerations of animal ethical studies and with the aim of primarily establishing the feasibility of CAD and 3D-printed guide plates during short-term observation. One day prior to the surgical experimentation, instruments including the surgical guide plates were sterilized under high temperature and pressure for 30 min and dried for 30 min. All 3 canines were shaved from the gluteal region up to the hock of the operated limb with care as not to cause skin injuries. During the operative day, preoperative Cefotaxime (3 mg/kg) was administered intravenously 30 min prior to the procedure for antibiotic prophylaxis. Sedation using Propofol (5 mg/kg) was also administered intravenously followed by endotracheal intubation and maintained using Propofol (0.5 mg/kg/min) and 3% Isoflurane (Inhaled). Following anesthesia, the experimental canines were placed in a left lateral decubitus position and the surgical site was disinfected three times using 5% povidone iodine + 75% alcohol.

An 8–10 cm incision was made and the location of the femoral bone was palpated and exposed through blunt dissection of the muscular septum. A Bovie was used to cauterize subcutaneous bleeding and dissect the deep fascia, the quadriceps femoris and expose the femoral bone. A 6–8 cm exposure of the femoral bone was made using a surgical blade to make an incision of the femoral periosteum, and a blunt dissection towards the posterior aspect of the adductor muscle group. The intramedullary nail was placed upon the exposed femoral bone and the mid-section of the implant was marked on the femoral bone using the Bovie. The surgical guide plate used in the study was then placed upon the femoral bone based on the mark as to prevent osteotomy on the area of the locking nails. A 1.5 mm Kirschner wire was used to fix the guide plate onto the surface of the femur and a 3 cm mid-diaphyseal femoral osteotomy was made using an oscillating saw. This was followed by reaming of the proximal and distal canal using a 4.5, 5.5 and 6.5 mm reamer. Proximal reaming was performed up to penetration of the pyriform fossa to allow insertion of the intramedullary rod. Using the surgical guide plate, all 6 locking nails were drilled onto the intramedullary rod both proximally and distally. An intraoperative radiograph was taken following insertion of all the implant components to complete the surgery. The incision was then washed with saline, sutured, disinfected using povidone iodine and packed.

**Fig. 5:**
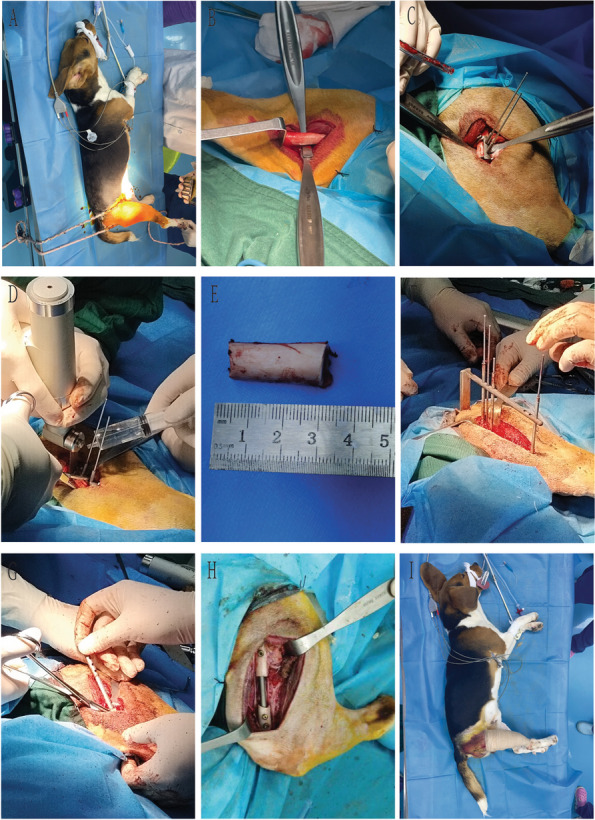
Surgical procedure: **A** Body positioning and limb fixation; **B** Exposure of the mid-femur; **C** Placement of the osteotomy guide plate; **D** Fixation the osteotomy guide plate; **E** Length of osteotomy; **F**/**G** Intramedullary nail implantation; **H** Completion of internal fixation; **I** Completed surgery with the incision packed and wrapped

Intraoperative vital signs and blood oxygenation was monitored throughout the surgery and postanesthetic recovery. All canines were given Meloxicam (0.2 mg/kg) and Cefquinome (2 mg/kg) intramuscularly for analgesic and infection prophylaxis, as well as a neck cone to prevent from licking the surgical incision. All 3 canines were monitored and recorded for the beginning of standing up and ambulation. The incision was monitored for infection, healing and sutures were removed in time. Postoperative radiographs were taken at 1, 2 and 3 months to assess fixation and euthanized at the end of 3 months by an intravenous injection of pentobarbital (430 mg/5 kg). A representation of the surgical procedure is shown in Fig. 5.

### Statistical analysis

All the obtained data were statistically analysed using SPSS 22.0 software (IBM, USA), and data description were expressed as mean ± standard deviation. In the femur model simulation experiment, the operation duration and the accuracy of locking nail placement in the two groups were evaluated using paired T-test.

## Results

### Study sample and 3D printed surgical instrument parameters

**Fig. 6:**
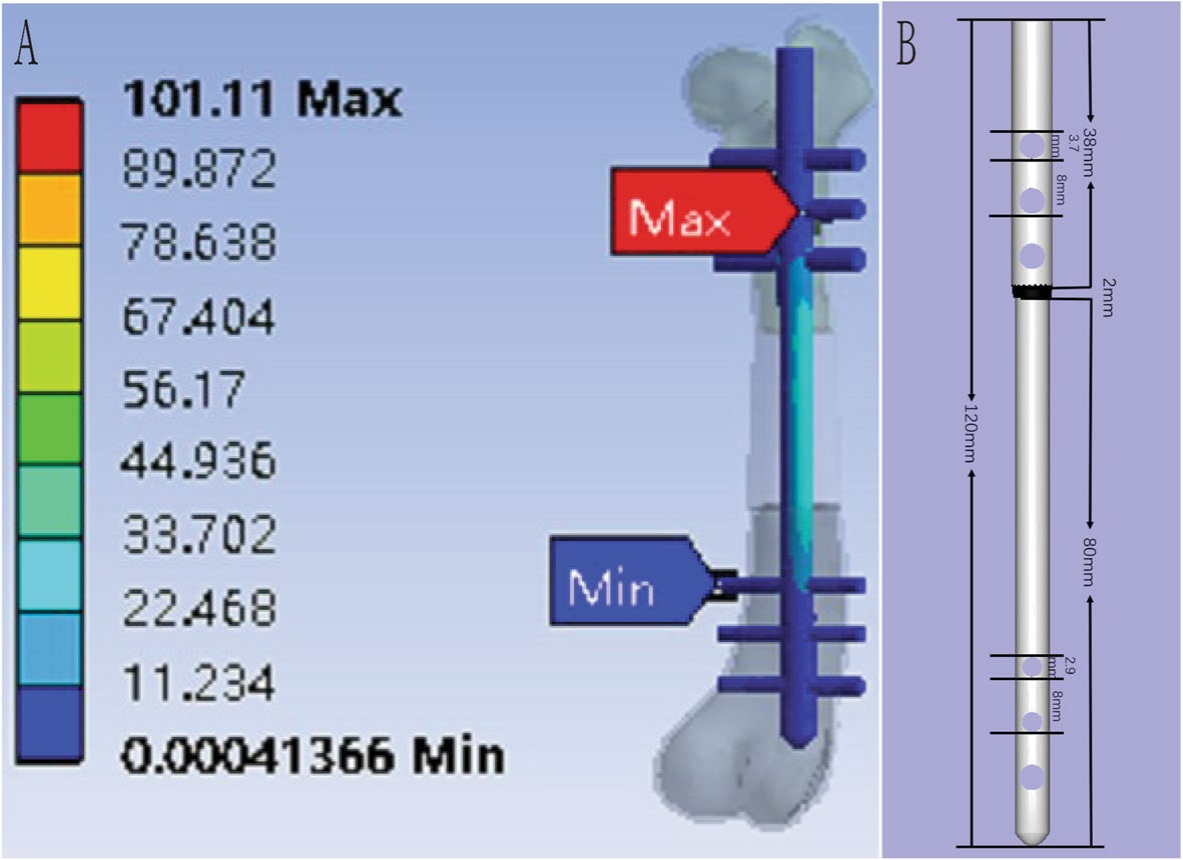
CAD-based intramedullary nail. **A** Result of FEA showing the weight distribution after osteotomy and intramedullary nail insertion. **B** Mechanical parameters of intramedullary nail

The mean femoral length, diameter of the medullary cavity and diameter of the mid-femur measured by CT scanning and three-dimensional reconstruction were 135.39 ± 6.09 (130.10–142.05) mm, 5.15 ± 0.34 (54.89–5.53) mm and 13.65 ± 1.67 (12.33–15.53) mm respectively. The measurements are summarized in Table 1. CAD was then used to construct a personalized intramedullary nail based on the femoral parameters. FEA results show that stress and strain were the largest at the distal end of the first locking nail and thus the intramedullary nail were designed as: total length of the intramedullary nail was designed to be 120 mm (proximal nail rod length = 38 mm and diameter = 6 mm, connecting joint length = 2 mm, distal nail rod length = 80 mm and diameter = 5.5 mm). All 3 locking nail orifice on both distal and proximal ends were equidistant to each other (8 mm). The distance between third proximal locking nail orifice to the first distal orifice was 60 mm. The total length of osteotomy was 30 mm, and the distance between the two nearest locking nail orifice was 15 mm. Diameter of the three proximal orifice was 3.7 mm (cortical tapping screw used = 3.5 mm in diameter), the first and second distal orifice are 2.9 mm (cortical tapping screw used = 2.7 mm in diameter), and the third orifice was 3.7 mm (cortical tapping screw used = 3.5 mm in diameter). This design was based on the existing screw parameters (Fig. 6).

**Table 1:** CAD measured femur parameters in Beagles

Number	Femur length	Diameter of medulla (mm)	Diameter of medial femur cortex (mm)
1	130.10	4.89	13.10
2	142.05	5.03	15.53
3	134.01	5.53	12.33
Mean ± SD	135.39 ± 6.09	5.15 ± 0.34	13.65 ± 1.67

### Simulated model fixation

**Fig. 7:**
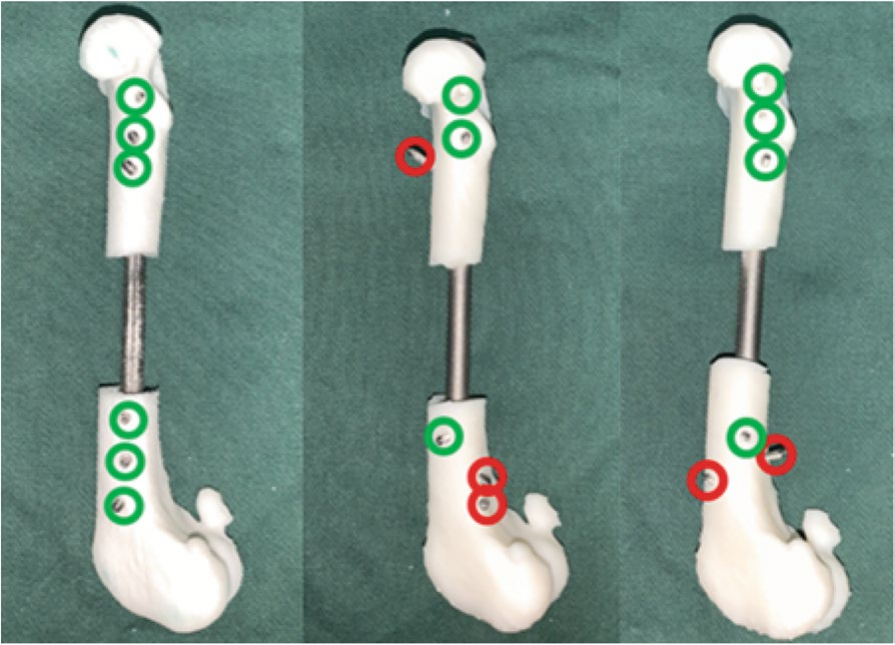
Accuracy measurement of screw placement (From left to right: model number 1, 11 and 13 shown in Table 2)

Our experimental results show that osteotomy and internal fixation could be accurately performed by utilizing personalized surgical instruments. A total of 60 screws were implanted in each group, with 59 screws accurately implanted by the CAD group, and 43 screws accurately implanted in the control group, reaching an accuracy of 98.3 ± 5.37 (83–100) % and 71.6 ± 23.6 (33.3–100) % for the experimental and control groups respectively, indicating a significant statistical difference between the two groups (*P* < 0.05). The average operation time was significantly shorter in the experimental group as compared to the control group (26 ± 4.6 (19–36) mins and 40.6 ± 14.1 (23–68) mins), and was assessed to be statistically significant (*P* < 0.05). In vitro experimental data is shown in Table 2, and the accuracy of the nail placement is shown in Fig. 7.

**Table 2:** In vitro experimental data showing the accuracy of each nail placement on the femur model during extracorporeal simulation. Accuracy of each nail is measured as a 1/6 of a whole percentage

Experimental Group			Control Group		
Model No.	Duration of operation (min)	Accuracy (%)	Model No.	Duration of operation (min)	Accuracy (%)
1	25	100	11	47	50
2	36	100	12	28	33.3
3	23	100	13	68	66.6
4	26	100	14	39	66.6
5	29	100	15	23	100
6	26	100	16	49	83.3
7	19	83	17	27	100
8	25	100	18	54	50
9	29	100	19	52	100
10	22	100	20	39	66.6

### In vivo segmental bone defect modelling in Beagles

**Fig. 8:**
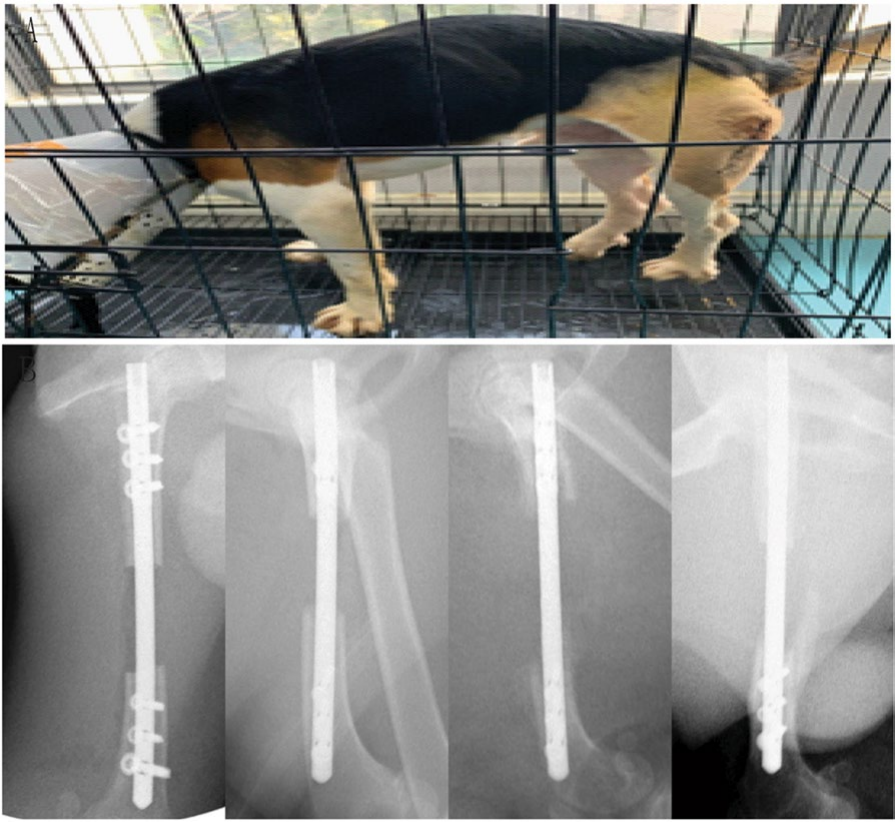
Postoperative conditions of the Beagle models showing: **A** Stable weight bearing on three limbs at 1 week; **B** X-ray radiographs postoperatively, and at 1, 2 and 3 months follow-up

All three Beagles in the study recovered to a regular oral diet post-surgery and survived at 3 months follow-up. All three Beagles were capable of standing with weight-bearing on three limbs postoperatively. The operative limb was capable of weight bearing at 2 weeks post-surgery and normal ambulation of the operated limb was recorded at 4 weeks. Redness and swelling, with effusion noticeable during the first three postoperative days and gradually decreased and fully recovered at 1-week post-surgery without any evidence of infection. Stitches were removed at 10–13 days after surgery accordingly to each incision. Fur growth around the surgical site was noticeable after 4 weeks post-surgery. X-ray radiographs taken on a monthly basis for three consecutive months showed no evidence of fracture of the intramedullary rod or loosening of the locking nails, showing satisfactory fixation (Fig. 8).

## Discussion

### Advantages of Beagles in a segmental bone defect model

Compared to canines, rodents are relatively easier to operate on and feed, but with obvious drawbacks of having different bone development processes from humans, and thus not optimal to reflect human bone defect diseases [13]. Large mammals as bone defect models are more reliable but with major drawbacks including a complex and difficult surgical procedure, low repeatability, feeding requirements, higher costs and stricter ethical implications [14, 15]. Existing studies on large animal bone defect models have used dogs (Beagles, Labradors), pigs (mini and micro pigs) and sheep with defects ranging from the skull and spine to long bones of the limbs [16, 17, 18]. Previous bone defects studies were mainly reported using partial cortex or borehole model of defect, with only few full-segment load-bearing bone defect model studies. Beagles were used to construct the segmental critical bone defect model in our study, mainly considering that canine femurs are structurally similar to human femurs. Beagle femurs also possess adequate bone volume for osteotomy and intramedullary nail fixation, which meets the requirements for the construction of segmental critical bone defect model anatomically. In this study, the length of the osteotomy was further lengthened according to each preoperatively measured femur. The conventional model requires a defect length 1.5 times the diameter of the bone, which is approximately 2.1 cm in length. In our study, we extended the length of osteotomy to 3 cm further emphasizing the changes in a pathological bone defect, while maintaining postoperative motor function. Beagles are also relatively social animals allowing for easier maintenance. Even within confinement, Beagles still maintain a large amount of activity which further promotes the recovery of postoperative limb function, strengthening the benefit of using Beagles in this particular type of study.

### Preoperative simulation in the construction of femoral bone defect model in Beagles

Previous studies based on preoperative simulation have reported the benefit of a significant increase in operative proficiency [18, 19]. Although there is a relative difference in material composition between in vitro and in vivo experimentation, a complete enactment of the surgical procedure can be achieved using 3D printed models allowing for a better understanding of each step [20]. In this study, construction of the 3D printed model based on CT scanning allows for a complete duplicate of the femur. In vitro emulation allows for simultaneous comprehensive understanding of the Beagle femoral structure as well as a solid practice of surgical technique. Osteotomy and intramedullary nail insertion during animal experimentation requires a degree of skills. The increased risk of failures for beginner researchers and the high costs required for each specimen further emphasizes the importance of preparation. Utilizing 3D printed models creates surgical conditions similar to real-time experimentation such as an accurate lateral incision of the quadriceps muscle to expose the femur for osteotomy. The medial side of the femur which is regarded as a blind spot during actual surgery can also be easily reconstructed with a surgical drape, exposing only the lateral half of the femoral model for experimentation. In this study, the entire simulation from osteotomy to internal fixation was performed by a constant group of researchers to minimize deviations in surgical techniques.

### CAD-constructed femoral defect model of Beagles

CAD, preoperative surgical emulation and use of auxiliary instruments has been recognized clinically and is reported to effectively increase surgical accuracy [21, 22]. In our study, CAD-based osteotomy guide plates and intramedullary nails were designed to ensure that all femoral osteotomy were consistent in length and conformed to the preoperative planning. The function of the 3D-printed instruments was verified by surgical simulation. Our results show that the usage of auxiliary instruments increased surgical efficiency, reflected by a significant decrease in surgical time and increase in the accuracy of nail placement. With CAD, repeated analysis of the surgical plan and potential hurdles during the operation process could be identified, allowing for gradual improvement. In one such situation, we found that the sensation of puncturing the bone cortex can be misleading as it only signifies the placement of the locking nail though two cortical layers but with not an accurate placement. We infer that is mainly due to the sensation determining the position of the nail but not the direction of insertion, commonly leading to the failure. This fortifies the need of an appropriate navigating equipment for positioning and orientation. Simultaneously, we noticed that locking nail failure mainly occurred at the distal end of osteotomy during simulation. This was mostly due to a difference in anatomical characteristic of the femur and the internal fixation apparatus (proximal end of the intramedullary nail thicker than the distal end, and the proximal end of the femoral bone marrow cavity thinner than the distal end) [23]. The press-fit between the intramedullary nail and the medullary cavity at the distal end of the femur was not as satisfactory as compared to the proximal end. Through analysis, extra care was allotted during the insertion and design of the intramedullary nail as to reduce the risk of implant failure.

FEA was also incorporated during the construction of the instruments, to further understand the overall stress distribution over the intramedullary nail. This record was the basis of the design for our intramedullary nails, having a diameter larger in proximal end where is stress concentrated, and consequent adjustments the thickness of different locking nails. This strengthens our findings showing that CAD allows for the constant improvement of surgical planning before actual animal experimentation.

### The role of 3D printing in the construction of femoral defect model in Beagles

Utilization of 3D printing is already involved in numerous clinical situations. At present, 3D printing has successfully been used to construct various personalized medical instruments, as well as tissues and organs for implantation, significantly contributing to the development of the medical industry [24, 25]. This report confirmed the feasibility of 3D printed personalized medical instruments in the future of precision medicine. A similar study by Saunders et al. [26] successfully constructed a segmental defect model of the femoral bone in dogs. But the breed of the specimens, the surgical plan and surgical instruments utilized were not introduced in detail, as well as a debatable short segment of bone defect, possibly leading to failure in future duplication. In our study, 3D printing was used throughout the entire study protocol. Surgical auxiliary instruments were printed with medical-grade titanium alloy powder and could be sterilized at high temperature to ensure sterility. The femur model and the surgical instruments were all constructed using 3D printing, allowing for a comprehensive examination of the operated bone in vitro, and preoperative simulation allowing for better familiarity of the surgical procedure. The surgical instruments effectively increased safety, accuracy and efficacy of the surgical procedure. Most importantly, the designed structure of the intramedullary nails played a major role in the postoperative recovery of the lower limb function after osteotomy. Without 3D printing, our study would have had major difficulties and would have presented with a relatively higher rate of failure. Construction of the critical bone defect model in a Beagle femur further confirms the importance of 3D printing for the preparation of medical instruments, as well as providing a feasible treatment plan for patients with bone defects unsuitable for common clinical strategies.

### Limitations and prospects

The large segmental bone defect model of the Beagle femur established in this part has successfully met the requirements of high repeatability and stabilization, but we do acknowledge the limitations to our study. Although the length of the bone defect can be fixed at 3 cm with the help of an osteotomy guide plate, the definite placement of the osteotomy could not be guaranteed to be uniform, potentially affecting fixation. Mechanical tests of torsion, lateral bending and tensile were also absent in the evaluation of the final results, resulting in a lack of proper quantification of the mechanical strength. The absence of a control group for In Vitro studies also may lead to bias in experimental results. In our future studies, we would aim to involve the arrangement of a control group during In Vitro experimentation using simplified instruments for comparison without compromising safety and further improve the analysis of the resulting mechanical strength to better evaluate stability.

## Conclusion

Through understanding and tailoring previous animal models of large segmental bone defects, our study combined CAD and 3D printing to construct a stable 3 cm segmental defect of the Beagle femoral model. CAD-based osteotomy guide plates and internal fixation instruments, shows potential for subsequent bone regeneration research, especially centred around weight-bearing bone tissue engineering. Utilization of CAD and 3D printing could provide assistance in complex procedures to improve overall efficacy and precision.

## Data Availability

No datasets were generated or analysed during the current study.
